# A kinetic model for quantitative evaluation of the effect of hydrogen and osmolarity on hydrogen production by *Caldicellulosiruptor saccharolyticus*

**DOI:** 10.1186/1754-6834-4-31

**Published:** 2011-09-13

**Authors:** Mattias Ljunggren, Karin Willquist, Guido Zacchi, Ed WJ van Niel

**Affiliations:** 1 Department of Chemical Engineering, Lund University, PO Box 123, SE-221 00 Lund, Sweden; 2 Department of Applied Microbiology, Lund University, PO Box 124, SE-221 00 Lund, Sweden

## Abstract

**Background:**

*Caldicellulosiruptor saccharolyticus *has attracted increased interest as an industrial hydrogen (H_2_) producer. The aim of the present study was to develop a kinetic growth model for this extreme thermophile. The model is based on Monod kinetics supplemented with the inhibitory effects of H_2 _and osmotic pressure, as well as the liquid-to-gas mass transfer of H_2_.

**Results:**

Mathematical expressions were developed to enable the simulation of microbial growth, substrate consumption and product formation. The model parameters were determined by fitting them to experimental data. The derived model corresponded well with experimental data from batch fermentations in which the stripping rates and substrate concentrations were varied. The model was used to simulate the inhibition of growth by H_2 _and solute concentrations, giving a critical dissolved H_2 _concentration of 2.2 mmol/L and an osmolarity of 0.27 to 29 mol/L. The inhibition by H_2_, being a function of the dissolved H_2 _concentration, was demonstrated to be mainly dependent on H_2 _productivity and mass transfer rate. The latter can be improved by increasing the stripping rate, thereby allowing higher H_2 _productivity. The experimentally determined degree of oversaturation of dissolved H_2 _was 12 to 34 times the equilibrium concentration and was comparable to the values given by the model.

**Conclusions:**

The derived model is the first mechanistically based model for fermentative H_2 _production and provides useful information to improve the understanding of the growth behavior of *C. saccharolyticus*. The model can be used to determine optimal operating conditions for H_2 _production regarding the substrate concentration and the stripping rate.

## Background

The question of climate change, together with the increasing scarcity and cost of fossil fuels, has triggered research on the sustainable production of energy carriers, such as biofuels. Although a wide spectrum of alternative fuels and processes are available, it is still not known which of them will succeed in replacing fossil fuels in the long term. Processes for producing some biofuels, such as ethanol and methane, are already highly developed for commercial use, while others, including biohydrogen, require more basic research before they can be produced in an economically feasible way [1].

The current major drawbacks of fermentative hydrogen (H_2_) production are its low yield [2] and the requirement of gas stripping to remove H_2 _from the liquid [3]. Dark fermentation can be performed at either moderate or elevated temperatures: the productivity is generally higher in the former case [4], whereas the latter provides higher yields [5]. However, for an economically sustainable biohydrogen process, high productivity should be accompanied by high yields [2]. One solution is to focus on the process design, such as the optimization of H_2 _removal and substrate concentration [6]. A higher substrate concentration is required to avoid excess amounts of water, since a surplus of process water has a negative effect on both the environmental and economic aspects of the process [7]. It is important to find alternatives to nitrogen (N_2_) sparging, as this dilutes the H_2_, leading to a lower-grade product or the need for expensive gas upgrading to remove the N_2_.

The extreme thermophile *Caldicellulosiruptor saccharolyticus *may be of industrial interest because of its ability to produce high yields of H_2 _from a wide variety of sugars [8,9], ranging from pentose and hexose monomers [10] to complex (hemi)cellulosic materials [8,11,12]. However, the growth of this microorganism, as well as the production of H_2 _and acetic acid, is subject to substrate and product inhibition [13]. In addition, it is sensitive to increased osmotic pressure [14], making it necessary to dilute the substrate. Moreover, *C. saccharolyticus *is inhibited by H_2 _[13], which is a general trait of H_2_-producing organisms. A high dissolved H_2 _concentration (H_2aq_) inhibits H_2_-generating hydrogenases [1,2], leading to increased nicotinamide adenine dinucleotide, reduced/nicotinamide adenine dinucleotide (NADH/NAD) ratios, resulting in a metabolic shift toward reduced products such as lactate and ethanol [1,3,4] and thus decreased H_2 _productivity.

The critical partial H_2 _pressure ($P_{H_{2}}$) is usually the parameter coupled to growth inhibition and lactate formation [5,13,15-17], although it has been shown that there are mass transfer limitations that cause the fermentation medium to be supersaturated with H_2 _[18,19]. Therefore, H_2aq _should be considered rather than $P_{H_{2}}$ when discussing inhibition by H_2_. Stripping with N_2_, which decreases H_2aq _[19], can significantly improve the H_2 _yield [3]. Indeed, optimizing the process should consist of a compromise between minimizing the stripping gas flow rate, henceforth called the "stripping rate," and maintaining a suitable H_2 _yield and productivity [20]. However, optimization must take H_2 _productivity into account, since it influences H_2aq _greatly; otherwise, the optimization will be relevant only to the specific system studied and cannot be applied to other biohydrogen systems. Furthermore, better insight into H_2 _productivity and liquid-to-gas mass transfer will help considerably in finding technical solutions allowing an economical and feasible fermentation process to be designed.

This paper describes the development of a kinetic Monod-based model for cell growth and product formation by *C. saccharolyticus*, which takes the liquid-to-gas mass transfer into consideration. The derived model successfully simulates batch fermentations of *C. saccharolyticus *and predicts the influence of H_2aq _and osmotic pressure on growth as well as the metabolic shift to lactate production. To the best of our knowledge, this is the first time the inhibitory effects and liquid-to-gas mass transfer of H_2 _and carbon dioxide (CO_2_) have been included in a model for fermentative H_2 _production. The results obtained with the model are relevant for biohydrogen systems other than that described herein using *C. saccharolyticus*.

## Methods

### Cultivation and fermentation

*C. saccharolyticus *was cultivated in a N_2 _atmosphere in modified DSM640 medium as described previously [9]. Cultures for inocula were grown overnight in 250-mL serum flasks containing 50 mL of modified DSM640 medium, together with a 0.4% carbon source and 0.02% cysteine.

Fermentations were performed in a jacketed 3-L reactor (Applikon, Schiedam, the Netherlands) at a working volume of 1 L. The pH was monitored using an ADI 1025 Bio Console Bio Controller (Applikon) and maintained at pH 6.6 (corresponding to neutral pH at 70°C) by the addition of sodium hydroxide (NaOH). The temperature was thermostatically maintained at 70°C ± 1°C, and the stirring rate was set to 350 rpm. Prior to inoculation, the medium was reduced by the addition of 0.1% cysteine. The fermentor was continuously stripped with N_2_.

A total of eight experiments were performed, with glucose concentrations of 5 or 10 g/L and stripping rates from 0.78 to 6 L of N_2_/hour, to estimate the parameters of the kinetic model. The experiments were performed on four different occasions. An overview of the experiments is given in Table 1. The H_2 _productivity and the cumulative H_2 _production were determined as described previously [21].

**Table 1:** Main characteristics of the eight fermentation experiments^a^

Experiment	Glucose concentration (g/L)	Stripping rate (L/hour)
1	5	6
2	10	6
3	5	6
4	5	0.78
5	5	6
6	5	1.2
7	5	1.56
8	10	6

^a^Experiments 3 and 4, as well as experiments 5 through 8, were performed using the inocula from the same preculture, respectively.

### Measurement of dissolved hydrogen concentration

The dissolved H_2 _concentration, H_2aq_, was determined in samples of cell suspensions from the bioreactor in serum flasks. The pressure in the flasks was reduced using a vacuum pump to withdraw the fermentation medium (30 to 40 mL) from the fermentor. The sealed flasks were placed in a water bath (20°C) with magnetic stirring to ensure good mixing. After one hour in the water bath, the pressure in the flasks was rapidly raised to atmospheric pressure by inserting a small needle through the cap, allowing air to enter the flasks. After 12 hours, the H_2 _concentration in the headspace of the serum flasks was measured as described below (Analytical methods). The volume of the collected liquid and the total volume of the individual serum flasks were measured to estimate the original H_2aq _in the sample.

### Analytical methods

Headspace samples were analyzed for CO_2 _and H_2 _using a Varian CP-4900 Micro GC gas chromatograph (Varian, Inc., Middelburg, the Netherlands) equipped with a thermal conductivity detector (100 mA). The results were analyzed using Galaxie Chromatography Workstation version 1.9.3.2 software (Varian, Inc.). The optical density of aerobic samples was measured at 620 nm using a spectrophotometer (Hitachi U-1100; Hitachi High-Technologies Corp., Tokyo, Japan). Concentrations of acetic acid, lactic acid and ethanol were analyzed using high-pressure liquid chromatography (Waters Corp., Milford, MA, USA). An Aminex HPX-87H ion exchange column (Bio-Rad Laboratories, Hercules, CA, USA) was used at 45°C with a mobile phase of 5 mM H_2_SO_4 _at a flow rate of 0.6 mL/minute. The chromatograph was equipped with a refractive index detector (RID-6A; Shimadzu, Kyoto, Japan).

### Modeling

The model employed in this study considers the kinetics of growth and product formation, including inhibition, liquid-to-gas mass transfer and the chemical equilibrium of CO_2 _and carbonates. The constants used in the model are listed in Table 2.

**Table 2:** The constants used in the model

Constant	Values
*K*_1_	6.3 atm
*K*_2_	10.3 L atm/K/mol
*K*_G_	0.048 mmol/L
$H_{H_{z}}$	7.40 × 10^-9 ^mol/L/Pa
$H_{CO_{z}}$	2.70 × 10^-7 ^mol/L/Pa
pH	6.6 L
*P*_tot_	1 atm
*R*	0.082 L atm/K/mol
*r*_cd_	0.014/hour
*T*	70°C
*V*_g_	0.05 L
*V*_i_	1.00 L

#### Growth and product kinetics in C. saccharolyticus

The kinetic model for *C. saccharolyticus *is based on simultaneous solution of the mass balance equations. The following reactions occurring in *C. saccharolyticus *were taken into consideration in the model:

(1) $$\text{Glucose}+2{\text{H}}_{2}\text{O}\to 2{\text{C}}_{\text{2}}{\text{H}}_{4}{\text{O}}_{\text{2}}+2\text{C}{\text{O}}_{2}+4{\text{H}}_{2}$$

(2) $$\text{Glucose}\to 2{\text{C}}_{3}{\text{H}}_{\text{6}}{\text{O}}_{\text{3}}$$

(3) $$\text{Glucose}\to k\;\text{C}{\text{H}}_{1.62}{\text{O}}_{0.46}{\text{N}}_{0.23}{\text{S}}_{0.0052}{\text{P}}_{0.0071}$$

The elemental composition of *C. saccharolyticus *was previously determined by de Vrije *et al*. [21]. The value of the stoichiometric parameter *k *in equation 3 is not known and was therefore estimated (described in detail below, Experimental design and estimation of kinetic parameters). Furthermore, reaction 3 is not balanced, since elements are available in the fermentation medium which were not considered in the model (for example, yeast extract). *C. saccharolyticus *is subject to several inhibiting agents affecting cell growth as well as the production of acetic acid and H_2_. These include (1) elevated concentrations of dissolved H_2 _and H_2aq_, and (2) high osmolarity. Osmolarity (named "OSM" in the model) is calculated by summing the molar can be described by the following expression:

(4) $$OSM=G+2\cdot Ac+2\cdot CO_{2sol}+2\cdot Lac+0.1$$,

where *G*, *Ac *and *Lac *are the concentrations of glucose, acetic acid and lactic acid, respectively, and CO_2sol _is the concentration of bicarbonate and carbonate, resulting from the CO_2 _produced by the bacterium. The stoichiometric factor 2 arises from the assumption that for each mole of acid produced, one mole of NaOH is added to maintain the pH. The background osmolarity, 0.10 mol/L, resulting from yeast extract and other nutrients was calculated based on osmolarity measurements presented previously [14].

The mass balance for the growth of the cells (in batch fermentation) can be described by the following expression:

(5) $$\text{Cell mass}:\;\frac{dX}{dt}=\left(\mu -r_{cd}\right)\;\cdot X$$,

where μ, *X *and *r*_cd _are the specific growth rate, the cell mass concentration and the cell death rate, respectively.

The specific growth rate, μ, is given by Monod kinetics:

(6) $$\mu =\mu_{max}\cdot \;\frac{G}{G+K_{G}}\cdot \left(1-{\left(\frac{OSM}{OSM_{crit}}\right)}^{n_{\mu }}\right)\cdot \left(1-{\left(\frac{H_{2aq}}{H_{2aqcrit}}\right)}^{n_{H_{2}}}\right)$$

Here μ_max _is the maximum specific growth rate, *G *is the glucose concentration and *K*_G _is the glucose affinity parameter. *OSM*_crit_, H_2aqcrit_,$n_{H_{2}}$ and *n*_μ _are the critical osmolarity, critical dissolved H_2 _concentration and the exponential parameters describing the level of inhibition, respectively. The critical parameters represent the values at which inhibition is 100%. The kinetic inhibition expressions, as presented by Han and Levenspiel [22], are valid only for concentrations of the inhibiting agents (OSM or H_2aq_) lower than the respective critical concentrations. Elevated osmolarity inhibits the growth of *C. saccharolyticus *[14], which may lead to a metabolic shift toward the production of lactic acid [23].

#### Product formation

The product formation rates were expressed using an adapted form of the Luedeking-Piret equation [24]:

(7) $$\frac{dP_{i}}{dt}=\alpha_{i}\cdot \mu \cdot X+\beta$$

Here, α is a growth-associated constant, and β is a non-growth-associated constant. All products were assumed to be only growth-associated, hence β is zero. This simplification is based on the assumption that other nutrients are in excess. It is further assumed that H_2 _inhibits its own production and thus also that of acetate in both a direct and an indirect way: indirectly through the inhibition of growth, and directly through specifically inhibiting the hydrogenases, which results in an increase in the NADH/NAD ratio in the cell [25]. This in turn induces the activity of the enzyme lactate dehydrogenase, initiating a metabolic shift toward lactate [23]. Besides being activated through H_2_, lactic acid production is also activated via osmolarity, which at high concentrations inhibits growth [23]. The growth-associated constants (equation 7) for the metabolic products are as follows:

(8) $$\alpha =R_{AcF}\cdot INHIB\;\;\;\text{mol}\;\text{Ac/(mol}\;\text{cells)}$$

(9) $$\alpha_{Lac}=R_{LacFH_{2}}\cdot ACT_{H_{2}}+{R_{Lac}}_{FOSM}\cdot ACT_{OSM}\;\;\text{mol Lac/(mol cells)}$$

(10) $$\alpha_{H_{2}}=\alpha_{Ac}\cdot \left(Y_{GH_{2}}∕Y_{GAc}\right)\;\;\text{mol}\;{\text{H}}_{\text{2}}∕\left(\text{mol}\;\text{cells}\right)$$

(11) $$\alpha_{CO_{2}}=\alpha_{H_{2}}\cdot \left(Y_{GCO_{2}}∕Y_{GH_{2}}\right)\;\;\text{mol}\;\text{C}{\text{O}}_{2}∕\left(\text{mol}\;\text{cells}\right)$$

Here *R*_AcF_, ${R_{Lac}}_{FH_{2}}$ and R_LacFOSM _are the factors relating the production rates of acetic acid and lactic acid to the growth rate; INHIB and ACT are the inhibition and activation factors, respectively (see below, Product formation) Y_GAc _and $Y_{GCO_{2}}$ are the stoichiometric yield coefficients of the reactions described above (equation 1); and $Y_{GH_{2}}$ is the estimated H_2 _yield coefficient (mol H_2_/mol glucose). The following inhibition mechanisms were studied.

Inhibition and activation by dissolved H_2_:

(12) $$INHIB=1-{\left(\frac{H_{2aq}}{{H_{2aq}}_{crit}}\right)}^{n_{H_{2}}}$$

(13) $$ACT_{H_{2}}={\left(\frac{H_{2aq}}{{H_{2aq}}_{crit}}\right)}^{n_{H_{2}}}\;$$

Activation of lactic acid production by osmolarity:

(14) $$ACT_{OSM}={\left(\frac{OSM}{OSM_{crit}}\right)}^{n\mu }$$

The mass balances of the products in the liquid phase consist of an accumulation, time-dependent, and a product formation rate, concentration-dependent, term, and, when applicable, a liquid-to-gas mass transfer term.

(15) $$\text{Acetic}\;\text{acid:}\;\;\;\frac{dA}{dt}=\alpha_{Ac}\cdot \mu \cdot X$$

(16) $$\text{Lactic}\;\text{acid:}\;\;\;\frac{dLac}{dt}=\alpha_{Lac}\cdot \mu \cdot X\;$$

(17) $${\text{H}}_{\text{2}}:\;\;\;\frac{dH_{2aq}}{dt}=\alpha_{H_{2}}\cdot \mu \cdot X-k_{l}a_{H_{2}}\cdot \left(H_{2aq}-H_{2aq}^{*}\right)$$

Here $K_{l}a_{H_{2}}$ is the overall volumetric mass transfer coefficient for H_2 _and $H_{2aq}^{*}$ is the H_2 _saturation concentration.

CO_2_:

(18) $$\begin{array}{ccc} \frac{dCO_{2aq}}{dt}={\alpha_{CO}}_{{}_{2}} & \cdot \mu \cdot X-k_{l}{a_{CO}}_{{}_{2}}\cdot \left(CO_{2aq}-CO_{2aq}^{*}\right)-CO_{2aq}\left(\frac{10^{-K_{1}}}{10^{-pH}}+10^{-K_{1}}\cdot \frac{10^{-K_{2}}}{{\left(10^{-pH}\right)}^{2}}\right) & \\ +CO_{2sol} & \end{array}$$

where $K_{l}a_{CO_{2}}$ is the overall volumetric mass transfer coefficient for CO_2_; CO_2aq _and $CO_{2aq}^{*}$ are the concentration of dissolved CO_2 _and the saturation concentration of CO_2_, respectively; *K*_1 _and *K*_2 _are the dissociation constants; and CO_2sol _is the total concentration of carbonates. The last two expressions in the CO_2 _mass balance (equation 18) are related to the reaction of CO_2 _with water to form carbonates (see Additional file: Liquid-to-gas mass transfer and chemistry of CO2__Theory_experiments_and_results). The expressions $K_{l}a_{H_{2}}\cdot \left(H_{2aq}-H_{2aq}^{*}\right)$ and $K_{l}a_{CO_{2}}\cdot \left(CO_{2aq}-CO_{2aq}^{*}\right)$ in equations 17 and 18 describe the liquid-to-gas mass transfer of H_2 _and CO_2_, respectively (see Additional file: Liquid-to-gas mass transfer and chemistry of CO2__Theory_experiments_and_results).

#### Substrate consumption

The mass balance of glucose can be written as follows:

(19) $$\frac{dG}{dt}=-\left[\left(1∕Y_{GX}\right)+\left(1∕Y_{GAc}\right)\cdot \alpha_{Ac}+\left(1∕Y_{GLac}\right)\cdot \alpha_{Lac}\right]\cdot \mu \cdot X$$,

where *Y*_GX _is the cell mass yield coefficient parameter (which is the model notation of the constant *k *in equation 3) and *Y*_GAc _and *Y*_GLac _are the stoichiometric yield coefficients (mol/mol substrate) describing the theoretical yields of cell mass, acetic acid and lactic acid, respectively.

#### Gas phase balances

The gaseous products are produced in the liquid phase, after which they are transported to the gas phase. The mass balances of the various gaseous compounds comprise an accumulation term and a gas-to-liquid mass transfer term as well as an inflow and an outflow.

(20) $${\text{H}}_{2}:\;\frac{dH_{2G}}{dt}=V_{l}∕V_{g}\cdot k_{l}a_{H_{2}}\cdot \left(H_{2aq}-H_{2aq}^{*}\right)-F_{out\;H_{2}}\cdot \frac{P_{tot}}{V_{g}\cdot R\cdot T}$$

(21) $$\text{C}{\text{O}}_{2}:\;\frac{dCO_{2G}}{dt}=V_{l}∕V_{g}\cdot k_{l}a_{CO_{2}}\cdot \left(CO_{2aq}-CO_{2aq}^{*}\right)-F_{out\;CO_{2}}\cdot \frac{P_{tot}}{V_{g}\cdot R\cdot T}$$

(22) $${\text{N}}_{2}:\;\frac{dN_{2}}{dt}=\left(F_{in\;N_{2}}-F_{out\;N_{2}}\right)\cdot \frac{P_{tot}}{V_{g}\cdot R\cdot T}$$

Here *V_l _*and *V*_g _are the liquid and gas volume; *k_l_a *is the volumetric mass transfer coefficient for the various compounds; H_2aq _and CO_2aq _are the dissolved concentrations of H_2 _and CO_2_; $H_{2aq}^{*}$ and $CO_{2aq}^{*}$ are the dissolved concentrations of the compounds at equilibrium; $F_{in\;N_{2}}$ is the flow rate of N_2 _into the fermentor; and $F_{out\;N_{2}}$, $F_{out\;CO_{2}}$ and $F_{out\;H_{2}}$ are the effluent flow rates of N_2_, CO_2 _and H_2_, respectively.

#### Other equations used

The equilibrium between CO_2_, bicarbonate and carbonate was taken into consideration as follows:

(23) $$\frac{dCO_{2sol}}{dt}=CO_{2aq}\left(\frac{10^{-K_{1}}}{10^{-pH}}+10^{-K_{1}}\cdot \frac{10^{-K_{2}}}{{\left(10^{-pH}\right)}^{2}}\right)-CO_{2sol}$$

Since the pH was controlled during fermentation, it was kept constant at 6.6 in the model. For more details, see Additional file 1: Liquid-to-gas mass transfer and chemistry of CO2__Theory_experiments_and_results.

Furthermore, the effect of the stripping rate on the volumetric mass transfer coefficients was determined by using the following equation:

(24) $$k_{l}a_{i}=k_{l}a_{i0}\cdot {\left(F_{in\;N_{2}}∕F_{0in\;N_{2}}\right)}^{\gamma }$$,

where *k_l_a_i0 _*is the volumetric mass transfer coefficient at a stripping rate of $F_{0in\;N_{2}}$, *k_l_a_i _*is the volumetric mass transfer coefficient at a stripping rate of $F_{in\;N_{2}}$ and γ is an experimentally determined exponential coefficient.

### Experimental design and estimation of kinetic parameters

The mass transfer parameters ($K_{l}a_{H_{2}}$ and $K_{l}a_{CO_{2}}$) and the exponential coefficient (γ) were determined before the parameters for the microbial kinetics were estimated. The mass transfer parameters were estimated using a methodology described by Hill [26]. The volumetric mass transfer coefficient for CO_2_, $K_{l}a_{CO_{2}}$, was determined and the volumetric mass transfer coefficient for H_2 _($K_{l}a_{H_{2}}$) was calculated using the following relation between *k_l_a *values and the diffusion coefficients for the compounds in water [18]:

(25) $$\frac{k_{l}a_{CO_{2}}}{k_{l}a_{H_{2}}}={\left(\frac{D_{CO_{2}}}{D_{H_{2}}}\right)}^{1∕2}$$

Here the diffusion coefficients for CO_2 _($D_{CO_{2}}$) and H_2 _($D_{H_{2}}$) are 1.98 × 10^-5 ^and 4.65 × 10^-5 ^cm^2^/second, respectively, according to Pauss *et al*. [18]. Only the most important results in the estimation of the mass transfer parameters are given here; the detailed results and the methodology can be found in Additional file: Liquid-to-gas mass transfer and chemistry of CO2__Theory_experiments_and_results.

To determine the kinetic parameters, fermentations were performed at two substrate concentrations and various stripping rates (Table 1). The kinetic parameters studied and estimated were *Y*_GX_, $Y_{GH_{2}}$, μ_max_, *R*_AcF_, ${R_{Lac}}_{FH_{2}}$, H_2aqcrit_, $n_{H_{2}}$, *R*_LacFOSM_, *OSM*_crit _and *n*_μ_. Since the experiments were characterized by different growth rates as well as H_2 _and biomass yields, all parameters except the three related to osmolarity (*R*_LacFOSM_, *OSM*_crit _and *n*_μ_) were estimated simultaneously for each of the 5 g/L experiments (that is, experiments 1 and 3 through 7), giving five parameter sets (Table 3). Experiments 6 and 7 were carried out under almost the same conditions with the same inocula and were thus used together to estimate one parameter set. The effect of osmolarity was assumed to be negligible in these experiments (experiments 1 and 3 through 7). The validity of this assumption was checked and is discussed below (Kinetic parameters: inhibition by osmolarity). An average of all parameters except μ_max _was then calculated and used to simulate all six experiments. The parameters related to osmolarity were estimated using the 10 g/L experiments (that is, experiments 2 and 8).

**Table 3:** The estimated model parameters for 5 g of glucose/L experimentsa

	Experiments									
	
	1		3		4		5		6 and 7	
	
Parameter	Value	95% (±)	Value	95% CI (±)	Value	95% CI (±)	Value	95% CI (±)	Value	95% CI (±)
$Y_{GH_{z}}$	6.0	0.2	5.1	0.2	3.6	0.1	4.5	0.1	4.71	0.13
*Y*_GX_	2.8	0.5	6.5	1.3	5.1	0.9	4.3	0.6	5.22	0.60
μ_max_	0.240	9 × 10^-3^	0.36	1.6 × 10^-2^	0.33	5.6 × 10^-2^	0.2	2 × 10^-4^	0.21	4.0 × 10^-3^
*R*_AcF_	1.4	0.2	2.0	0.2	2.3	0.4	1.8	2 × 10^-2^	2.0	8 × 10^-2^
*R*_LacF_	0.36	7 × 10^-2^	0.11	9 × 10^-2^	0.37	9 × 10^-2^	9 × 10^-2^	0.22	0.17	5 × 10^-3^
H_2aqcrit_	1.9 × 10^-3^	4 × 10^-4^	1.8 × 10^-3^	3 × 10^-4^	2.3 × 10^-3^	7 × 10^-4^	1.3 × 10^-3^	2 × 10^-4^	2.7 × 10^-3^	5 × 10^-4^
$n_{H_{z}}$	10.7	5.6	3.1	0.6	3.3	0.8	5.2	1.7	2.9	0.3

^a^We assumed that osmolarity did not have an effect at this low concentration. The validity of the assumption was checked and discussed in the Kinetic parameters: inhibition by osmolarity section of text. The value of the parameter and the 95% confidence interval (95% CI) are given for each parameter.

Finally, the cell death rate (*r*_cd_; equation 5), was calculated directly from the experimental data by determining, for each experiment, the slope of the biomass curve as it declined and calculating an average value of the slope based on all experiments. The overall affinity constant (*K*_G_; equation 6) was taken from the literature on bacteria with the same type of glucose transporter system, that is, the ATP-binding cassette transport system (values are given in Table 2) [10,27,28].

The parameters in the model were estimated using a nonlinear least squares method which minimizes the objective function:

(26) $$LSQ=\sum_{i=component}\left[{\sum_{t=time}\left(Y_{exp,i,t}-Y_{model,i,t}\right)}^{2}\right]$$,

where LSQ is the least squares quadratic, that is, the sum of the squared residuals, *Y*_exp,*i*,*t *_and *Y*_model,*i*,*t*_, are the experimental and model values, respectively, at time *t *for each compound *i*, that is, glucose, acetic acid, lactic acid, cell mass and H_2_. The parameter estimations were carried out and the 95% confidence intervals were calculated using the MATLAB functions *nlinfit *and *nlparci*, respectively (MathWorks, Natick, MA, USA).

## Results and Discussion

The order in which the parameters are estimated is important. Since the mass transfer influences fermentation, and thus the corresponding kinetic parameters, the mass transfer parameters were estimated prior to determining the fermentation-related kinetic parameters.

### Estimation of the volumetric mass transfer coefficient

The estimated overall volumetric mass transfer coefficients for CO_2 _($K_{l}a_{CO_{2}}$) in the fermentation medium were in the range of 3.4 to 7.2/hour for stripping rates ranging from 2.0 to 9.8 L/hour (33 to 164 mL/minute). The $K_{l}a_{CO_{2}}$ values obtained with their respective stripping rates were used to calculate the exponential parameter γ (equation 24), giving a value of 0.46 ± 0.02, which agrees well with previous findings [20,29]. On the basis of the mass transfer results of CO_2 _and equation 25 $K_{l}a_{H_{2}}$ was determined to be 9.0 ± 0.1/hour at a stripping rate of 6 L/hour (100 mL/minute). This value is consistent with previous results for similar systems with stripping rates between 5 and 2,000 mL/minute, where the *k_l_a *values were in the range of 1 to 120/hour [18,20]. Hence, the expression calculating $K_{l}a_{H_{2}}$ (equation 24) is:

(27) $$k_{l}a_{H_{2}}=9.0\cdot {\left(F_{in\;N_{2}}∕6.0\right)}^{0.46}$$,

where $F_{in\;N_{2}}$ is the stripping rate. For more detailed results on mass transfer, see Additional file: Liquid-to-gas mass transfer and chemistry of CO2__Theory_experiments_and_results.

### Fermentation

In fermentation experiments 1 through 7, the glucose was completely consumed and acetic acid, H_2 _and lactic acid were the main products (Figures 1, 2, 3, 4, 5, 6 and 7). In experiment 8, complete glucose consumption was not achieved within 40 hours (Figure 8).

**Figure 1:**
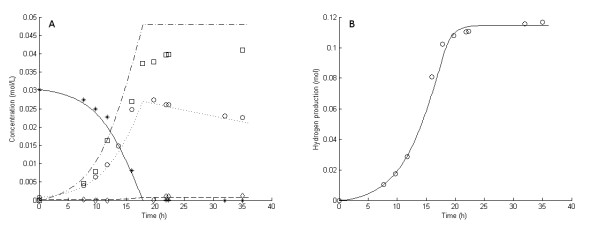
**Experimental and model results for experiment 1**. **Experimental results: (A) **Liquid concentrations: Glucose (asterisks), acetic acid (open squares), cell mass (open circles), lactic acid (open diamonds). **(B) **Accumulated hydrogen (H_2_) production (open circles). **Model results: **Average values were used for all parameters except μ_max_. **(A) **Liquid concentrations: Glucose (solid lines), acetic acid (dashed-dotted lines), cell mass (dotted lines), lactic acid (dashed lines). **(B) **Accumulated H_2 _production (straight lines).

**Figure 2:**
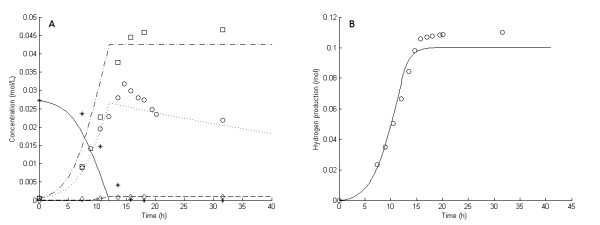
**Experimental and model results for experiment 3**. **Experimental results: (A) **Liquid concentrations: Glucose (asterisks), acetic acid (open squares), cell mass (open circles), lactic acid (open diamonds). **(B) **Accumulated H_2 _production (open circles). **Model results: **Average values were used for all parameters except μ_max_. **(A) **Liquid concentrations: Glucose (solid lines), acetic acid (dashed-dotted lines), cell mass (dotted lines), lactic acid (dashed lines). **(B) **Accumulated H_2 _production (straight lines).

**Figure 3:**
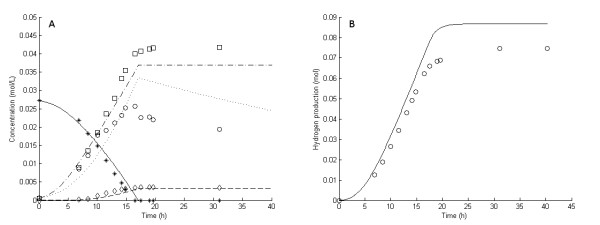
**Experimental and model results for experiment 4**. **Experimental results: (A) **Liquid concentrations: Glucose (asterisks), acetic acid (open squares), cell mass (open circles), lactic acid (open diamonds). **(B) **Accumulated H_2 _production (open circles). **Model results: **Average values were used for all parameters except μ_max_. **(A) **Liquid concentrations: Glucose (solid lines), acetic acid (dashed-dotted lines), cell mass (dotted lines), lactic acid (dashed lines). **(B) **Accumulated H_2 _production (straight lines).

**Figure 4:**
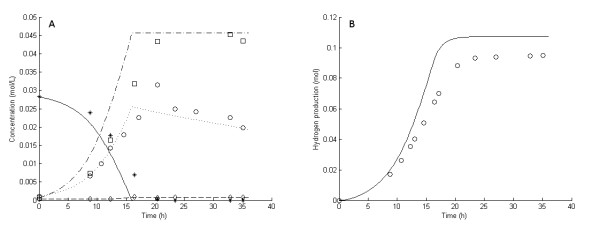
**Experimental and model results for experiment 5**. **Experimental results: (A) **Liquid concentrations: Glucose (asterisks), acetic acid (open squares), cell mass (open circles), lactic acid (open diamonds). **(B) **Accumulated H_2 _production (open circles). **Model results: **Average values were used for all parameters except μ_max_. **(A) **Liquid concentrations: Glucose (solid lines), acetic acid (dashed-dotted lines), cell mass (dotted lines), lactic acid (dashed lines). **(B) **Accumulated H_2 _production (straight lines).

**Figure 5:**
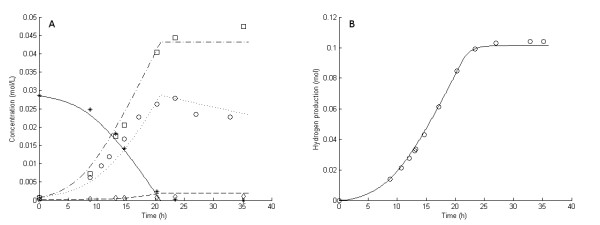
**Experimental and model results for experiment 6**. **Experimental results: (A) **Liquid concentrations: Glucose (asterisks), acetic acid (open squares), cell mass (open circles), lactic acid (open diamonds). **(B) **Accumulated H_2 _production (open circles). **Model results: **Average values were used for all parameters except μ_max_. **(A) **Liquid concentrations: Glucose (solid lines), acetic acid (dashed-dotted lines), cell mass (dotted lines), lactic acid (dashed lines). **(B) **Accumulated H_2 _production (straight lines).

**Figure 6:**
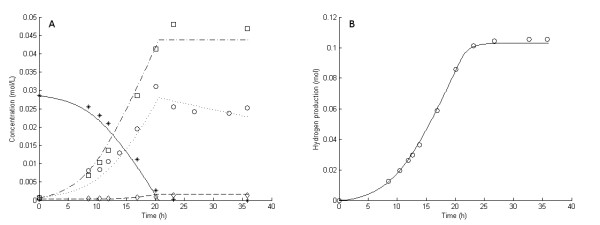
**Experimental and model results for experiment 7**. **Experimental results: (A) **Liquid concentrations: Glucose (asterisks), acetic acid (open squares), cell mass (open circles), lactic acid (open diamonds). **(B) **Accumulated H_2 _production (open circles). **Model results: **Average values were used for all parameters except μ_max_. **(A) **Liquid concentrations: Glucose (solid lines), acetic acid (dashed-dotted lines), cell mass (dotted lines), lactic acid (dashed lines). **(B) **Accumulated H_2 _production (straight lines).

**Figure 7:**
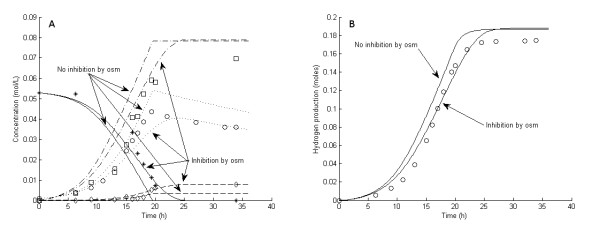
**Experimental and model results for experiment 2**. **Experimental results: (A) **Liquid concentrations: Glucose (asterisks), acetic acid (open squares), cell mass (open circles), lactic acid (open diamonds). **(B) **Accumulated H_2 _production (open circles). **Model results: **Average values were used for all parameters except μ_max_. For μ_max_, a value of 0.25/hour was used. **(A) **Liquid concentrations: Glucose (solid lines), acetic acid (dashed-dotted lines), cell mass (dotted lines), lactic acid (dashed lines). **(B) **Accumulated H_2 _production (straight lines).

**Figure 8:**
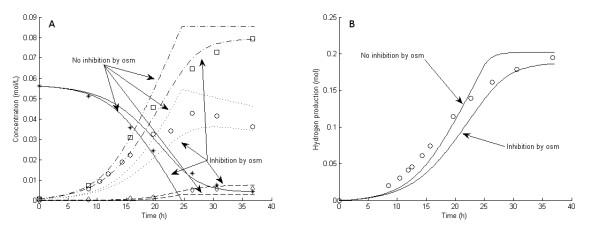
**Experimental and model results for experiment 8**. **Experimental results: (A) **Liquid concentrations: Glucose (asterisks), acetic acid (open squares), cell mass (open circles), lactic acid (open diamonds). **(B) **Accumulated H_2 _production (open circles). **Model results: **Average values were used for all parameters except μ_max_. For μ_max_, the average growth rate of experiment 5 through 7 was used (0.22/hour). **(A) **Liquid concentrations: Glucose (solid lines), acetic acid (dashed-dotted lines), cell mass (dotted lines), lactic acid (dashed lines). **(B) **Accumulated H_2 _production (straight lines).

The six 5 g/L experiments (that is, experiments 1 and 3 through 7) differed with respect to growth rate and H_2 _yield (Figures 1, 2, 3, 4, 5 and 6). The reason for the variations is not clear. However, the two sets of experiments, carried out in parallel using inocula from the same preculture (experiments 3 and 4 as well as experiments 5 through 8), showed a strong resemblance. Batch experiments are generally characterized by poor reproducibility, as has also been observed with *Escherichia coli *[30]. Owing to the variations between the experiments, each of the 5 g/L experiments was used separately to estimate all parameters except *OSM*_crit_, *n*_μ _and *R*_LacFOSM_, which were estimated using the 10 g/L experiments (experiments 2 and 8 and Figures 7 and 8). In addition, acetic acid and H_2 _were not produced at a stoichiometric ratio (1:2) in any of the experiments, which is in agreement with prior observations in continuous culture [15,21] where the H_2_/acetic acid ratio depended on the dilution rate. Instead, less acetate was produced in relation to H_2_, and the H_2 _yield was thus considered a parameter in the model. A possible explanation of the nonstoichiometric yield is that acetic acid is used as a building block by the cells.

### Growth rates

The estimated maximum growth rate of *C. saccharolyticus *varied between the different experiments; however, the values were similar for experiments carried out in parallel (that is, experiments 3 and 4 and 5 through 8) (Table 3). The μ_max _determined for glucose ranged from 0.21 to 0.36/hour and corresponded well with earlier findings, which varied from 0.2 to 0.5/hour [14,21]. Vrije *et al*. [21] found different values of μ_max _obtained in washout experiments with *C. saccharolyticus*, depending on the growth history of the culture. The reason for the variation in growth rates has not yet been elucidated, but the quality of the inocula used seems to depend on its growth history. The variation in growth rate was considered in the present model by not using an average value of μ_max_, while average values were used for all the other parameters (described in more detail below, Kinteic parameters: inhibition by dissolved hydrogen).

Together with changes in the product yields, the variations in growth rates strongly suggest that an as yet unknown factor influences the metabolism. Also, it is likely that the highest value of μ_max _determined in this study (0.36/hour) is not the true maximum, that is to say, the real μ_max _that can be achieved for glucose by this organism. This could mean that the growth of *C. saccharolyticus *is normally suppressed, as has been observed for *E. coli *[30]. The cause of the suppression of the growth of *C. saccharolyticus *is not understood and must be further studied to gain a better understanding of the physiology of the microorganism and to improve the model [21].

### Kinetic parameters: inhibition by dissolved hydrogen

As described above (Growth and product kinetics in C. *saccharolyticus*), the effects of two environmental conditions, that is to say, H_2aq _and osmolarity, on the growth, the production of acetic acid and H_2_, and the activation of lactic acid production of *C. saccharolyticus *were studied. All parameters except and *R*_LacFOSM_, *OSM*_crit _and *n*_μ _were estimated for each of the experiments with 5 g/L glucose (Table 3), from which their average values were calculated (Table 4). The experimental results are presented in Figures 1, 2, 3, 4, 5, and 6, together with the predictions of the model using the average values of the parameters (except for μ_max_). The average value of μ_max _was not used because of the large variation in this parameter; instead, the average within each set of parallel experiments was used. The model then agreed well with the experimental results and also reflected the effect of H_2aq _on the microorganism. Interestingly, the parameter $n_{H_{2}}$ has quite a high value (4.5), which indicates a steplike response to the inhibiting agent, H_2_; that is, at lower concentrations, the inhibition increases only a little with the concentration, whereas at higher concentrations the inhibition increases dramatically with increased concentration. The value of the H_2 _yield coefficient parameter, $Y_{GH_{2}}$, ranged from 3.7 to 6.2 mol H_2_/mol glucose. The upper value exceeds the Thauer limit, which is 4 mol H_2_/mol glucose [31]. However, the observed H_2 _yield obtained in the simulations and in the experiments never exceeded the Thauer limit; that is, the molar ratio of H_2 _produced to glucose consumed is always below 4. The high values of $Y_{GH_{2}}$ are an effect of the nonstoichiometric ratio between H_2 _and acetic acid seen during the experiments, described as $Y_{GH_{2}}∕Y_{GAc}$ in the model (equation 10). The ratios are often above 2 mol H_2_/mol acetic acid and, in the model, this comes out as a higher H_2 _yield coefficient parameter. The reason for the H_2 _and acetic acid ratio not being equal to 2, as the stoichiometry of reaction 1 shows, might be that some of the acetic acid produced is taken up and utilized by the cell.

**Table 4:** The average, maximum and minimum values of the parameters based on the confidence intervals^a^

Parameter	Mean	Minimum	Maximum
$Y_{GH_{z}}$	4.77 mol/mol	3.46 mol/mol	6.19 mol/mol
*Y_GX_*	4.78 mol/mol	2.30 mol/mol	7.76 mol/mol
μ_max_	0.28/hour	0.21/hour	0.51/hour
*R_AcF_*	1.91 hour/hour	1.24 hour/hour	3.40 hour/hour
*R_LAcF_*	0.20 hour/hour	0.13 hour/hour	0.43 hour/hour
*H*_2*aqcrit*_	2.2 × 10^-3 ^mol/L	9.6 × 10^-4 ^mol/L	4.1 × 10^-3 ^mol/L
$n_{H_{z}}$	4.50	0.84	16.24

^a^The values given are based on experiments 1, 3, 4, 5, 6 and 7.

### Dissolved hydrogen: degree of supersaturation and its effect

**Figure 9:**
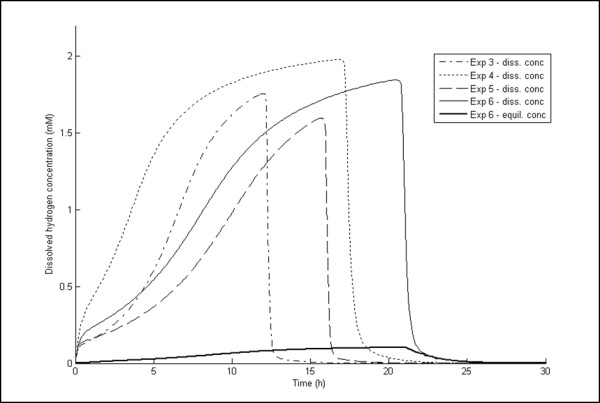
**Dissolved hydrogen concentration as a function of fermentation time**. The dissolved H_2 _concentration predicted by the model as a function of fermentation time for experiments 3, 4, 5 and 6 is shown. Note the rapid increase in dissolved H_2 _concentration in experiment 4, which is the effect of the low stripping rate and high growth rate. The predicted equilibrium dissolved H_2 _concentration (based on the partial pressure of H_2_) is also included for experiment 6.

Supersaturation of dissolved H_2 _occurred during fermentation, as measured experimentally and predicted by the model (Table 5), as reported previously [18,19]. Oversaturation ranged from 4.9 to 52 times the saturation concentration, which corresponds well with the results of other studies [18,19]. The large deviation in the measured values of H_2aq _(Table 5) is an effect of the large uncertainties in the measurements. The oversaturation of H_2aq _can also be clearly seen in Figure 9, where values of H_2aq _calculated with the model for a few experiments are shown together with the saturation concentration of H_2 _for experiment 6.

**Table 5:** The measured dissolved hydrogen concentration under different fermentation conditions compared with the model^a^

Experiment	5	6	7	8
H_2 _concentration	3.0%	9.1%	7.5%	3.6%
H_2 _productivity	8.4 mmol/L/hour	6.0 mmol/L/hour	6.2 mmol/L/hour	10.7 mmol/L/hour
H_2aq_	0.28 ± 0.01 mmol/L	0.8 ± 0.5 mmol/L	0.34 ± 0.02 mmol/L	0.9 ± 0.5 mmol/L
H_2aq _at equilibrium	0.022 mmol/L	0.069 mmol/L	0.056 mmol/L	0.027 mmol/L
Oversaturation	12.5 ± 0.3	12 ± 7	12.5 ± 0.4	34 ± 19
H_2aq _(model)	0.96 mmol/L	1.50 mmol/L	1.36 mmol/L	1.22 mmol/L

^a^Hydrogen (H_2_) concentration, H_2 _productivity and H_2aq _are the experimentally determined H_2 _concentration in the gas phase, H_2 _productivity and dissolved H_2 _concentration, respectively. H_2aq _at equilibrium is the corresponding theoretical H_2aq _at equilibrium with the gas. Oversaturation is the experimentally determined degree of H_2 _oversaturation in the liquid. H_2aq _(model) is the dissolved H_2 _concentration calculated by the model under the same conditions.

The experiment with the lowest stripping rate (experiment 4) showed the highest production of lactic acid (Figure 3). The other two experiments with low stripping rates (experiments 6 and 7) (Figures 5 and 6) showed only a slight increase in lactic acid production compared to the experiments with high stripping rates (experiments 1, 3 and 5) (Figures 1, 2 and 4) and very little lactic acid production compared to experiment 4. This can be explained by the lower growth rates resulting in lower H_2 _productivity, and hence lower values of H_2aq_, in experiments 5 through 7 (Figures 4, 5 and 6). Experiments 3 and 4 both showed high initial growth rates characterized by a high value of μ_max _and hence high H_2 _productivity, leading to higher values of H_2aq_. In experiment 4, with a low stripping rate (0.78 L/hour), H_2aq _reached a plateau faster than it did in the other experiments (Figure 9). At that stage, the culture reached maximum H_2 _productivity (data not shown), which resulted in linear rates of glucose consumption and H_2 _and acetic acid production (Figure 4). This behavior was reflected by the model (Figure 4) and can be explained by growth inhibition by H_2_. When H_2 _productivity reaches a critical value in the fermentation system, the cells respond with a reduction in the specific growth rate, and hence a plateau is observed. This plateau was reached much faster in experiment 4 than in the other two low-stripping-rate experiments (experiments 6 and 7), which was due to a combination of the higher initial growth rate and H_2 _productivity and the lower stripping rate (Table 1).

**Figure 10:**
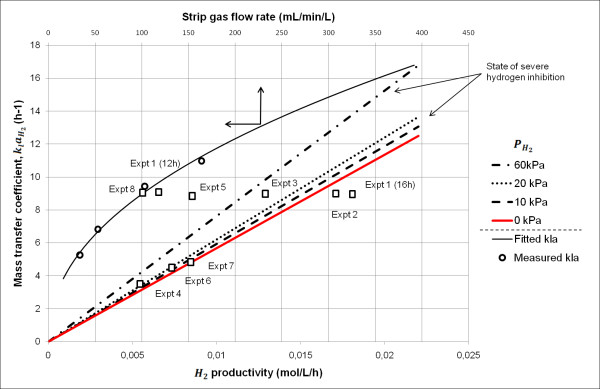
**State of severe hydrogen inhibition as a function of hydrogen productivity, $P_{H_{2}}$ and $k_{l}a_{H_{2}}$**. The straight lines show the combinations of H_2 _productivity, $P_{H_{2}}$ and $K_{l}a_{H_{2}}$, resulting in a state of severe H_2 _inhibition. The state of severe H_2 _inhibition is assumed to occur at a value of H_2aq _equal to 80% of H_2aqcrit _(corresponding to a reduction in the growth and H_2 _production rate of 37%). The further away from, and above, the lines the state of a fermentation experiment is, the lower the inhibition by H_2_. An increase in H_2 _productivity and a decrease in $K_{l}a_{H_{2}}$ move the state of the fermentation to the right and down, respectively, on the graph, while an increase in $P_{H_{2}}$ increases the slope of the lines. These changes all increase the dissolved H_2 _concentration and move the state of a fermentation experiment closer to a state of H_2 _inhibition. The open squares show the state of each experiment at the time of maximum productivity. An additional point is included for experiment 1 (12 hours), showing the state four hours before maximum productivity occurred. The curved line shows the relation between the stripping rate and $K_{l}a_{H_{2}}$ (the relation given by equation 27). The experimental $K_{l}a_{H_{2}}$ values (calculated from experimental values of $K_{l}a_{CO_{2}}$ using equation 25) are also included (open circles).

The model with estimated parameters for inhibition by H_2aq _can be used to indicate how H_2 _productivity, $P_{H_{2}}$, H_2aq _and the overall mass transfer coefficient are related (Figure 10). The dissolved H_2 _concentration in experiment 4 reached almost 80% of the value of H_2aqcrit _(indicated by the red line in Figure 10) after 10 hours, and, Dissolved hydrogen: degree of supersaturation and its effect), H_2 _productivity and hence H_2aq _were constant until glucose was depleted (Figures 3 and 9). The dissolved H_2 _concentration in experiment 5, on the other hand, was always well above the red line in Figure 10 and also showed little lactic acid production. In experiments 6 and 7, H_2aq _also ended up near the red line (Figure 10); however, both reached it much later (at 17 hours; that is, only 4 hours before the glucose was depleted). This was due to lower growth rates and slightly higher stripping rates. Finally, H_2aq _in experiment 1 crossed the red line after 16 hours with little production of lactic acid, which may be due to an exponential increase in H_2 _productivity, and the line was crossed when very little glucose remained; hence almost no substrate was left for lactic acid production (Figure 1).

The organism thus seems to respond to an increase in H_2aq _by adjusting its growth rate and directing its metabolism toward the production of lactate, thus fine-tuning H_2 _productivity to prevent H_2aq _values detrimental to the cell. On the basis of both the experimental data and the model, it is clear that dissolved H_2 _has a negative effect on *C. saccharolyticus *and that H_2 _productivity and the stripping rate have a considerable impact on H_2aq_. This can explain why fermentation with high H_2 _productivity commonly results in low H_2 _yields [15,21,32,33] and why an increase in the stripping rate tends to increase H_2 _yield and productivity [20].

### Predicting the effect of dissolved hydrogen concentration

**Figure 11:**
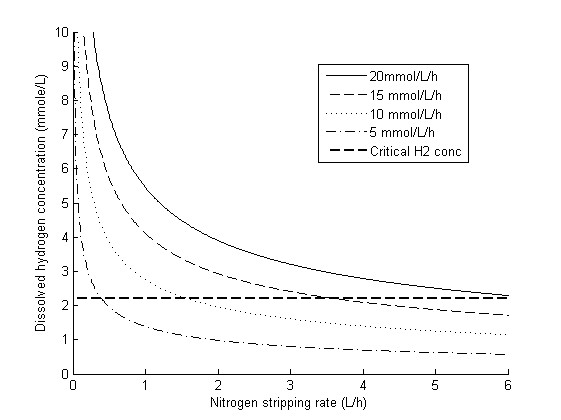
**H_2aq _as a function of the stripping rate and hydrogen productivity**. The horizontal line shows the estimated critical H_2 _concentration.

The dissolved H_2 _concentration can be lowered by stripping with N_2 _gas (Figure 11), which also results in higher H_2 _yields [19,34-36]. Kraemer and Bagley [20] published details of a study in which they optimized the stripping rate with respect to H_2 _yield and productivity. They concluded that a stripping rate of 12 mL/minute, resulting in a volumetric mass transfer rate of 5/hour, was optimal. However, this was only valid for their particular system and not for systems with higher H_2 _productivity and different liquid-to-gas mass transfer properties. Figure 11 shows H_2aq _calculated by our model at various stripping rates and H_2 _productivities for the reactor system used herein. It is evident that H_2aq _is a function of both variables.

Furthermore, the current model cannot simulate a system without sparging, since *k_l_a *is based on the stripping rate (equation 24). However, Figure 10 provides an excellent basis for further discussions, since the relation between $K_{l}a_{H_{2}}$, $P_{H_{2}}$ and H_2 _productivity still holds, even without stripping. For instance, with *C. saccharolyticus *at a total pressure of 1 atm, no stripping, a productivity of 10 mmol H_2_/L/hour, and a gas composition of 60% H_2 _and 40% CO_2_, a value of $K_{l}a_{H_{2}}$ of at least 7.5/hour is required to avoid serious H_2 _inhibition (Figure 10).

### Kinetic parameters: inhibition by osmolarity

Experiments 2 and 8 were carried out with almost identical initial conditions, but they progressed quite differently, probably due mainly to different inocula and a slight difference in the initial glucose concentration. It was evident that H_2aq _as the sole inhibitor could not adequately describe the system at elevated substrate concentrations (Figures 7 and 8). Indeed, the model fitted the data from experiments 2 and 8 very well after introducing growth inhibition by osmotic pressure.

The average value of μ_max _in experiments 5 through 7, which were carried out using the same inocula as experiment 8, was used for experiment 8. However, since no experiments were carried out using the same inocula as experiment 2, together with a low glucose concentration, the value of μ_max _for experiment 2 is not known. Instead, the average value of μ_max _for the three sets of 5 g/L experiments (experiment 1, experiments 3 and 4, and experiments 5 through 7) was assumed (0.27/hour) when estimating the inhibition parameters for osmolarity, that is, *R*_LacFOSM_, *OSM*_crit _and *n*_μ _(Table 6). Applying this extended model to the experiments with lower glucose concentrations confirmed that the osmolarity had a minor influence on the outcomes of the fermentations under those conditions. To estimate the three parameters describing the inhibition by osmolarity, μ_max _for experiment 2 had to be assumed. This assumption mainly affects the value of the exponential factor, *n*_μ_, and not *R*_LacFOSM _or *OSM*_crit _(Table 5). This strongly indicates that *OSM*_crit _for *C. saccharolyticus *is in the range of 0.27 to 0.29 mol/L, a range that is in agreement with previous results of a study by Willquist *et al*. [14].

**Table 6:** The effect of the assumption of μ_max _for experiment 2 on the estimates of the parameters associated with osmolarity^a^

Experiment 2 μ_max_	0.27/hour	0.24/hour	0.30/hour
R_LacFOSM_	0.41 (0.22)	0.54 (0.32)	0.34 (0.25)
OSM_crit_	0.28 (0.01)	0.27 (0.01)	0.29 (0.01)
*n*_μ_	4.68 (0.75)	8.73 (2.23)	3.61 (0.67)

^a^Data are values of the parameter with the confidence interval (CI, 95%) are given for each parameter.

Although this rather simple model describes the effect of osmolarity well, some aspects of it cannot be reproduced by this type of unstructured, mechanistic model. It is evident from Figure 8 that the growth rate, and hence the glucose consumption and acetic acid production rates, are not affected at the initiation of growth, since the model excluding inhibition by osmolarity fits the first three points perfectly. However, at an osmolarity of about 0.2 mol/L, there is a shift toward a reduction in the growth rate, glucose consumption, and acetic acid production rate, as well as an increase in lactic acid production, rapidly at first, followed by a slow decline of all of them. It is not possible to model this effect, that is, a rapid shift followed by a slow gradual decrease, using a simple inhibition expression. One could assume a very high exponential factor, but that would predict only the beginning of the experiment; however, as inhibition came into effect, the decrease in the growth rate to zero would not be slow and gradual, but very considerable and rapid.

## Conclusions

Batch fermentations of *C. saccharolyticus *on glucose were successfully simulated using Monod kinetics extended to include liquid-to-gas mass transfer and inhibition by H_2aq _and osmolarity. In agreement with previous measurements [18,19], the model predicted high oversaturation of H_2 _in the liquid, which was also confirmed experimentally.

To the best of our knowledge, we have demonstrated for the first time the possibility of predicting H_2aq _and its effect on the fermentation in a laboratory-scale bioreactor as a function of stripping rate and productivity using the derived model. With this tool, it was possible to conclude that the inhibition of growth and H_2 _production depend on both H_2aq _and osmolarity. The model described the inhibition by both H_2aq _and osmolarity satisfactorily and can hence be used to predict the optimal stripping rate and substrate concentration. It also shows how *C. saccharolyticus *responds to increased osmolarity and H_2aq_. In addition, the modeled relationship between mass transfer, H_2 _productivity and H_2aq _is not specific to *C. saccharolyticus*, but can also be applied to other systems. To widen the applicability of the model even further, future studies should aim at incorporating the effect of the stirrer speed and the absence of stripping on the liquid-to-gas mass transfer.

### Nomenclature

Ac: acetic acid concentration (mol/L); CO_2aq_: CO_2 _concentration in the liquid phase (mol/L); $CO_{2aq}^{*}$: saturation concentration (mol/L); CO_2sol_: solubilized CO_2 _(mol/L); $F_{out\;CO_{2}}$: flow rate of CO_2 _out of the fermentor (L/hour); $F_{out\;H_{2}}$: flow rate of H_2 _out of the fermentor (L/hour); $F_{in\;N_{2}}$: flow rate of N_2 _into the fermentor (L/hour); $F_{out\;N_{2}}$: flow rate of N_2 _out of the fermentor (L/hour); *G*: substrate concentration (mol/L); H_2aq_: H_2 _concentration in the liquid phase (mol/L); H_2aqcrit_: critical H_2 _concentration in the liquid phase (mol/L); $H_{2aq}^{*}$: saturation concentration (mol/L); $H_{H_{2}}$: Henry's constant for H_2 _(L atm/mol); $H_{CO_{2}}$: Henry's constant for CO_2 _(L atm/mol); *K*_1_: dissociation constant for CO_2 _into bicarbonate; K_2_: dissociation constant for bicarbonate into carbonate; $K_{l}a_{H_{2}}$: mass transfer coefficient for H_2 _(per hour); $K_{l}a_{CO_{2}}$: mass transfer coefficient for CO_2 _(per hour); *K*_G_: saturation constant (mol/L); Lac: lactic acid concentration (mol/L); *n*_μ_: inhibition coefficient for inhibition by osmolarity; $n_{H_{2}}$: inhibition coefficient for inhibition by H_2_; *OSM*: osmolarity parameter (mol/L); *OSM*_crit_: critical osmolarity parameter (mol/L); $P_{H_{2}}$: partial pressure of H_2 _(Pa); $p_{H_{2}crit}$: critical partial pressure of H_2 _(Pa); *P*_tot_: total pressure (Pa); PPi: pyrophosphate; LSQ: least squares quadratic; *R*: gas constant (L atm/K/mol); *R*_AcF_: maximum Ac production rate (mol/g cell mass/hour); ${R_{Lac}}_{FH_{2}}$: maximum Lac production rate caused by H_2 _(mol/g cell mass/hour); *R*_LacFOSM_: maximum Lac production rate caused by osmotic pressure (mol/g cell mass/hour); *r*_cd_: death rate of cells (per hour); *T*: temperature (Kelvin); *V*_g_: gas volume in liquid (L); *V*_l_: liquid volume in reactor (L); *X*: cell mass concentration (mol/L); *Y*_GAc_: stoichiometric acetic acid yield (mol acetic acid/mol substrate); $Y_{GCO_{2}}$: stoichiometric CO_2 _yield (mol CO_2_/mol substrate); $Y_{GH_{2}}$: stoichiometric H_2 _yield (mol H_2_/mol substrate); *Y*_GLac_: stoichiometric lactic acid yield (mol lactic acid/mol substrate); *Y*_GX_: biomass yield (mol cell mass/mol substrate); α: cell-growth-associated constant; α_Ac_: cell-growth-associated constant for Ac production rate (mol Ac/mol cells); $\alpha_{CO_{2}}$: cell growth-associated constant for CO_2 _production rate (mol CO_2_/mol cells); $\alpha_{H_{2}}$: cell growth-associated constant for H_2 _production rate (mol H_2_/mol cells); α_Lac_: cell-growth-associated constant for Lac production rate (mol Lac/mol cells); β: non-cell-growth-associated constant; γ: exponential coefficient for mass transfer correlation; μ: specific growth rate (per hour); μ_max_: maximum specific growth rate (per hour).

## Competing interests

The authors declare that they have no competing interests.

## Authors' contributions

ML developed the model, carried out the parameter estimations and planned as well as performed the mass transfer experiments. ML also wrote the main part of the manuscript and took part in the planning and execution of the fermentation experiments. KW took part in the development of the model, planned and carried out the main part of the fermentation experiments, analyzed the results and assisted in the mass transfer experiments. KW also wrote parts of the manuscript. GZ and EVN participated in the coordination of the study and reviewed the manuscript. All authors read and approved the final manuscript.

## Supplementary Material

Additional file 1 **Liquid-to-gas mass transfer and chemistry of CO2__Theory_experiments_and_results**. This file contains information on the estimation of the mass transfer parameters as well as the set-up of the mass transfer experiments. The file also contains more detailed information concerning the theory behind the chemistry of carbon dioxide and liquid-to-gas mass transfer. Click here for file
