# Supplementary material for: A kinetic model for quantitative evaluation of the effect of hydrogen and osmolarity on hydrogen production by Caldicellulosiruptor saccharolyticus
Source: Biotechnol Biofuels. 2011 Sep 13;4:31. doi: 10.1186/1754-6834-4-31 (PMC3236304; doi:10.1186/1754-6834-4-31)
Supplement: Additional file 1 — Liquid-to-gas mass transfer and chemistry of CO2__Theory_experiments_and_results. This file contains information on the estimation of the mass transfer parameters as well as the set-up of the mass transfer experiments. The file also contains more detailed information concerning the theory behind the chemistry of carbon dioxide and liquid-to-gas mass transfer. [file 1754-6834-4-31-S1.DOCX]

Additional file

## Liquid-to-gas mass transfer and chemistry of CO_2_: Theory, experiments and results

This file presents additional material not included in the main article, and describes the aqueous chemistry of CO_2_, and the two-film theory of mass transfer, as well as the experimental work performed, the mass-transfer parameter estimation and results.

# Theory

## The chemistry of carbon dioxide

Carbon dioxide is a fermentation product which reacts with water forming bicarbonate (HCO_3_^-^) and carbonate (CO_3_^-2^) in pH-dependent reactions. The equilibrium reactions of carbon dioxide, bicarbonate, and carbonate are therefore included in the proposed model:

${CO}_{2aq}+H_{2}O \leftrightarrow H^{+}+H{CO}_{3}^{-}$ $K_{1}=\frac{\left[ H^{+} \right]\left[ H{CO}_{3}^{-} \right]}{\left[ {CO}_{2aq} \right]}$ (S-1)

$H{CO}_{3}^{-}\leftrightarrow H^{+}+{CO}_{3}^{2-}$ $K_{2}=\frac{\left[ H^{+} \right]\left[ {CO}_{3}^{2-} \right]}{\left[ H{CO}_{3}^{-} \right]}$ (S-2)

Here, $K_{1}$ and $K_{2}$ are the dissociation constants of reaction S-1 and S-2, respectively. The molar concentration of bicarbonate and carbonate can be described as follows.

${HCO}_{3}^{-}={CO}_{2aq}\cdot\left( \frac{{10}^{-K_{1}}}{{10}^{-pH}} \right)$ (S-3)

${CO}_{3}^{2-}={HCO}_{3}^{-}\cdot\left( \frac{{10}^{-K_{2}}}{{10}^{-pH}} \right)$ (S-4)

The sum of all CO_2_ ionic species (${HCO}_{3}^{-}$ and ${CO}_{3}^{2-}$), ${CO}_{2sol}$, can be expressed as in the following equation.

${CO}_{2sol}={CO}_{2aq}\cdot\left( \frac{{10}^{-K_{1}}}{{10}^{-pH}}+{10}^{-K_{1}}\cdot\frac{{10}^{-K_{2}}}{\left( {10}^{-pH} \right)^{2}} \right)$ (S-5)

The bicarbonate and carbonate reactions are described by differential equations.

$\frac{d{HCO}_{3}^{-}}{dt}={CO}_{2aq}\cdot\left( \frac{{10}^{-K_{1}}}{{10}^{-pH}} \right){-HCO}_{3}^{-}$ (S-6)

$\frac{d{CO}_{3}^{2-}}{dt} ={HCO}_{3}^{-}\cdot\left( \frac{{10}^{-K_{2}}}{{10}^{-pH}} \right)-{CO}_{3}^{2-}$ (S-7)

Similarly, ${CO}_{2sol}$ is described by the following equation.

$\frac{d{CO}_{2sol}}{dt}={CO}_{2aq}\cdot\left( \frac{{10}^{-K_{1}}}{{10}^{-pH}}+{10}^{-K_{1}}\cdot\frac{{10}^{-K_{2}}}{\left( {10}^{-pH} \right)^{2}} \right)-{CO}_{2sol}$ (S-8)

However, the reactions are considered to be in equilibrium at all times, which means that the reactions have infinitely fast reaction rates, i.e. $\frac{d{HCO}_{3}^{-}}{dt}$, $\frac{d{CO}_{3}^{2-}}{dt}$ and $\frac{d{CO}_{2sol}}{dt}$ are equal to zero.

The pH of the system is described by the following equation:

$pH= -log\left( {10}^{-{pH}_{0}}+{HCO}_{3}^{-} \right)$ (S-9)

where the influence of ${CO}_{3}^{2-}$ is neglected, due to the neutral to acidic conditions, which result in very small carbonate concentrations, leading to very small effects on the H^+^ concentration. ${pH}_{0}$ is the pH at the start of the experiment.

## Effects of buffering medium on pH and mass transfer

The fermentation medium is buffering, which means that the change in pH when stripping with 50% CO_2_ will be much smaller in the fermentation medium than in pure water. Hence, the value of $K_{1}$ in pure water (10^-6.3^, at 25ºC) cannot be used and the value must be estimated. However, this changes the amount of ${HCO}_{3}^{-}$ in the fermentation medium, and would cause an underestimation of the CO_2_ transported into the medium. To circumvent this, $K_{2}$ was estimated so as to correctly describe the total amount of bicarbonate and carbonate in the fermentation medium. $K_{2}$ was determined by performing titration experiments. Hence, ${CO}_{3}^{2-}$ does not actually represent carbonate, but carbonate and a considerable part of the bicarbonate as well, i.e. it is a “pseudo” concentration.

## Correlation between mass transfer and gas flow rate

In this system, hydrogen and carbon dioxide are produced in the liquid phase and are then transported into the gas phase. The rate of transport determines the accumulation of the gaseous products in the liquid phase, which can inhibit the microorganism.

The two-film theory was used to estimate the concentrations of the dissolved gases. In this theory, the mass-transfer rate is described by a concentration difference (a linear driving force) and a mass-transfer coefficient:

$mass transfer=k_{l}a\cdot\left( c_{A}^{*}-c_{A} \right)$ $k_{l}a_{H_{2}}\cdot\left( H_{2aq}-H_{2aq}^{*} \right)$ (S-10)

where $k_{l}a$ is the overall volumetric mass-transfer coefficient, $c_{A}^{*}$ $c_{A}^{*}$ is the saturation concentration in the bulk liquid, and $c_{A}$ $c_{A}$ is the actual concentration in the bulk liquid. The numerical values of these parameters depend on the compounds and the liquid solution. The parameter $c_{A}^{*}$ is calculated using Henry’s law and the partial pressure of the gas in the gas bulk phase, together with Henry’s constant for this gas.

Since $k_{l}a$ varies with the rate of gas flow through the reactor, it is important to know the extent to which this changes in order to be able to simulate the various stripping rates. A parameter called the superficial gas velocity, $u_{g}$ (m/s), is often applied:

$u_{g}=F/A$ (S-11)

where A is the reactor’s cross sectional area (m^2^) and F is the total gas flow rate (m^3^/h). Moreover, $k_{l}a$ is a function of $u_{g}$:

$k_{l}a=ku_{g}^{\gamma}$ (S-12)

where $\gamma$ is an exponential coefficient ranging between 0.4 and 0.7, and $k$ is a constant depending on the reactor setup and the physical properties of the liquid medium. If the reactor setup and liquid medium are not altered in the fermentation experiments, the following relation can be obtained:

$k_{l}a_{{CO}_{2}}=k_{l}a_{{CO}_{2}0}\cdot\left( F/{F_{0}} \right)^{\gamma}$ (S-13)

where $k_{l}a_{{CO}_{2}0}$ is the volumetric mass-transfer coefficient at a gas flow rate of $F_{0}$, and $k_{l}a_{{CO}_{2}}$ is the volumetric mass-transfer coefficient at a gas flow rate of F.

# Materials and methods

## Experimental setup

The buffering capacity of the medium was determined by titration experiments, which were performed by titrating 100 mL of the autoclaved culture medium with 10 wt% HCl. The amount of HCl added and the p H were recorded.

The volumetric mass-transfer coefficient ($k_{l}a$) of carbon dioxide was determined by stripping a liquid with pure nitrogen and a mixture of nitrogen and carbon dioxide, and measuring the change in pH. Since $k_{l}a$ depends on the medium, mass-transfer experiments were carried out using autoclaved fermentation medium, with dead cells at an optical density of 1. The mass-transfer experiments were performed in a jacketed 3-L reactor (Applikon, Schiedam, the Netherlands) at 70ºC with a working volume of 1 L (i.e., the same type of reactor and under the same conditions as in the fermentation experiments). Gas was flushed through the reactor; first pure nitrogen and then a mixture of 50% carbon dioxide and 50% nitrogen. When the pH had stabilized, the liquid was again stripped with pure nitrogen. The mass-transfer rates were studied at four different stripping rates: 33.5, 53, 103 and 164 mL/min. The pH was recorded continuously. Furthermore, the overhead space of the liquid was flushed with additional nitrogen to reduce the effect of headspace CO_2_ interacting with the liquid. This is important at low stripping rates when headspace CO_2_ significantly decreases the effect of stripping. The total gas flow in the fermentor headspace during stripping was kept constant at 260 mL/min. The sparging of the headspace is motivated by the fact that the concentrations of carbon dioxide and hydrogen in the headspace never exceeded 8% and 13%, respectively, during fermentation.

## Parameter estimation

The parameters were estimated in the following order: first $K_{1}$, then $K_{2}$ and lastly $k_{l}a_{{CO}_{2}0}$ and $\gamma$. $K_{1}$ was determined to set the minimum pH of each experiment, then $K_{2}$ was determined to give the correct amount of CO_2_ transferred into the fermentation medium. Finally, the mass-transfer parameters were estimated.

The estimations were performed using a non-linear least-squares method (*nlinfit*, MatLab^®^, MathWorks™) and the confidence intervals (95%) were calculated with the MatLab function *nlparci*.

**Results of the mass-transfer experiments**

The estimated values of $K_{1}$ and $K_{2}$ were 10.68 ± 0.04 and 2.13, respectively. Figure S-1 shows the experimental results together with the modeled results. The model overestimated the rate of pH change at the beginning and end of all the experiments (Figures S-1 and S-2).

***Figure S-1.*** *The pseudo CO3^2-^ concentration: experimental (—*—) and simulated (—). The experimental results were obtained by combining titration results with CO_2_ flushing results.*

The reason for this is probably that the model assumes ideal mixing in the liquid and gas phases in the reactor. This is a simplification which is mainly observed as equilibrium is approached. The mass-transfer rate was determined during stripping of the medium with N_2_ at different stripping rates, as this best represents fermentation (the fermentation medium is at least saturated with hydrogen) and the effects of non-idealities are minimized. The mass-transfer coefficient was estimated using the first 10-15 minutes of stripping to further minimize the effects of non-idealities. The results are given in Table S-1. The left column gives $k_{l}a_{{CO}_{2}}$ values where the overall mass-transfer coefficient at each gas flow rate was estimated separately using a least-squares method. The right column gives values where the parameters $k_{l}a_{{CO}_{2}0}$ and $\gamma$ in equation S-13 were estimated using all four experiments with

***
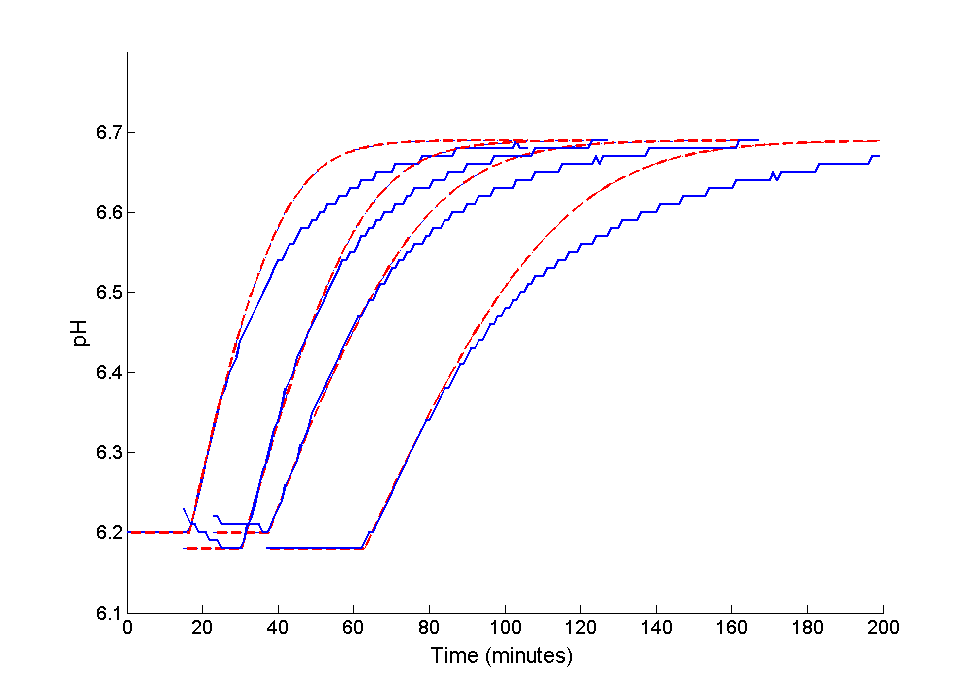
Figure S-2.*** *The change in pH when stripping the medium with pure N_2_ at (from left to right): 33.5, 53, 103 and 164 mL/min at 70ºC in a jacketed 3-L reactor (Applikon, Schiedam, the Netherlands). Measured pH (——), modeled pH (------).*

different gas flow rates. When $F_{0}$ was set to 100 mL/min, $k_{l}a_{{CO}_{2}0}$ and $\gamma$ were estimated to be 5.85 ± 0.07 h^-1^ and 0.46 ± 0.02, respectively. These results are very reasonable. In a previous study on a similar system with gas flow rates between 200 and 2000 mL/min, the values of $k_{l}a$ were determined to lie in the range of 20-120 h^-1^ (Pauss et al.).

*Table S-1. The table gives the estimated overall mass-transfer coefficients and the confidence interval (CI) for CO_2_. The left column gives values where each* $k_{l}a_{{CO}_{2}}$ *value was estimated separately. The right column gives the* $k_{l}a_{{CO}_{2}}$ *values calculated using Eq. S-13, and where* $k_{l}a_{{CO}_{2}0}$ *and* $\gamma$ *were fitted to all four data sets simultaneously.*

| **Stripping gas flow rate**  **(mL/min)** | $\boldsymbol{k}_{\boldsymbol{l}}\boldsymbol{a}_{\boldsymbol{CO}_{\boldsymbol{2}}}$ **(CI)**  **(h^-1^)** | $\boldsymbol{k}_{\boldsymbol{l}}\boldsymbol{a}_{\boldsymbol{CO}_{\boldsymbol{2}}}$ **(Eq. S-13)**  **(h^-1^)** |
| --- | --- | --- |
| **33.5** | 3.43 (0.04) | 3.54 |
| **53** | 4.46 (0.12) | 4.37 |
| **103** | 6.16 (0.12) | 5.93 |
| **164** | 7.17 (0.09) | 7.33 |
